# Serum soluble (pro)renin receptor level as a prognostic factor in patients undergoing maintenance hemodialysis

**DOI:** 10.1038/s41598-021-96892-6

**Published:** 2021-08-31

**Authors:** Yoshifumi Amari, Satoshi Morimoto, Chikahito Suda, Takeshi Iida, Hidenobu Okuda, Takatomi Yurugi, Yasuo Oyama, Naoki Aoyama, Fumitaka Nakajima, Atsuhiro Ichihara

**Affiliations:** 1 Department of Nephrology, Moriguchi Keijinkai Hospital, Osaka, Japan; 2 Department of Endocrinology and Hypertension, Tokyo Women’s Medical University, 8-1 Kawada-cho, Shinjuku-ku, Tokyo, 162-8666 Japan; 3 Department of Nephrology and Dialysis, Neyagawa Keijinkai Clinic, Osaka, Japan; 4 Department of Nephrology and Dialysis, Moriguchi Keijinkai Clinic, Osaka, Japan; 5 Department of Nephrology and Dialysis, Kadoma Keijinkai Clinic, Osaka, Japan

**Keywords:** Medical research, Nephrology

## Abstract

The (pro)renin receptor [(P)RR)] is a multifunctional protein that is cleaved to generate the soluble (P)RR [s(P)RR], reflecting the status of the tissue renin-angiotensin system and/or activity of the (P)RR. The serum s(P)RR level is associated with arteriosclerosis, independent of other risk factors, in patients undergoing hemodialysis (HD). This study was conducted to investigate whether the s(P)RR level was associated with new-onset cardiovascular events or malignant diseases and poor prognosis in patients undergoing HD. Overall, 258 patients [70 (61–76) years, 146 males] undergoing maintenance HD were prospectively followed up for 60 months. We investigated the relationships between s(P)RR levels and new-onset cardiovascular events/ malignant diseases and mortality during the follow-up period using Cox proportional hazard analyses. The cumulative incidence of new-onset cardiovascular events (*P* = 0.009) and deaths (*P* < 0.001), but not of malignant diseases, was significantly greater in patients with higher serum s(P)RR level (≥ 29.8 ng/ml) than in those with lower s(P)RR level (< 29.8 ng/ml). A high serum s(P)RR level was independently correlated with cardiovascular mortality (95% CI 1.001–1.083, *P* = 0.046). The serum s(P)RR level was associated with cardiovascular events and mortality, thus qualifying as a biomarker for identifying patients requiring intensive care.

## Introduction

The (pro)renin receptor [(P)RR], a specific receptor for renin and prorenin, consists of 350 amino acids with a single transmembrane domain and preferentially binds to renin and prorenin^1^. It is widely expressed in various organs, such as the brain, heart, and kidneys^2,3^. Non-proteolytic renin activation by the binding of prorenin to the extracellular domain of (P)RR^4^ accelerates the conversion of angiotensinogen to angiotensin (Ang) I. This mechanism has been proposed as a source of renin activity in the tissue renin-angiotensin system^1^. The soluble form of PRR[s(P)RR] is generated by intracellular cleavage by processing enzymes and is secreted into the extracellular space and found in the blood^5^. These findings suggest that s(P)RR can serve as a biomarker that reflects the status of the tissue RAS and/or (P)RR activity^6,7^. Moreover, (P)RR is a multi-functioning protein that allows local production of Ang I from angiotensinogen and induces intracellular signals independent of RAS activation.

It was recently discovered that (P)RR also functions as an adaptor protein between the Wnt receptor complex and vacuolar proton-translocating adenosine triphosphatase (V-ATPase)^8^. (P)RR- and V-ATPase-mediated acidification was shown to be essential for the Wnt/β-signaling pathway^8^, which is implicated in stem-cell biology and human diseases, including cancer, and has important roles in embryonic development, such as in axis formation and the patterning of the central nervous system^9–11^. Recently, accumulating evidence has revealed that overexpression of (P)RR, which may contribute to cancer initiation and progression, has been observed in various cancers including breast carcinoma^12^, pancreatic ductal adenocarcinoma^13^, glioma^14^, and aldosterone-producing adenoma^15^. Patients undergoing hemodialysis (HD) have a poor prognosis because of the increased prevalence of cardiovascular diseases (CVD) in this population^16,17^. Patients with heart failures have significantly higher plasma s(P)RR levels than control subjects^18^. We previously reported that the serum s(P)RR level was associated with arteriosclerosis, independent of other risk factors, in patients undergoing HD^19^, and the high serum s(P)RR level was associated with increases in brain natriuretic peptide (BNP), a marker of left ventricular dysfunction^20^, independent of other risk factors. These suggested that the increased expression of (P)RR may be associated with the progression of heart failure in patients undergoing HD^21^. Furthermore, it has been reported that patients undergoing HD are at a high risk for malignant diseases^22,23^. However, whether the blood s(P)RR levels are associated with the development of cardiovascular events or malignant diseases in patients undergoing HD has not been reported. Furthermore, it remains undetermined whether the blood s(P)RR level is related to total deaths and/or cardiovascular deaths in patients undergoing HD. On the basis of these background findings and unresolved questions, the present study aimed to investigate whether the serum s(P)RR level was associated with new-onset cardiovascular events or malignant diseases and with prognosis in patients undergoing HD during a follow-up period of 60 months.

## Methods

### Study subjects

The participants were outpatients on maintenance HD at the Kadoma Keijinnkai Clinic, Neyagawa Keijinnkai Clinic, and Moriguchi Keijinnkai Clinic in Osaka Prefecture, Japan, in April 2014. All three clinics are affiliated with the Moriguchi Keijinkai Hospital, Osaka, Japan. This study was approved by the ethics committee of Tokyo Women’s Medical University (approval number: 2703) and was conducted in accordance with principles of the 1975 Declaration of Helsinki, as revised in 2013. All patients were enrolled after they provided written informed consent. A total of 258 patients undergoing maintenance HD, who were enrolled in our previous cohort study that investigated the relationships between serum s(P)RR levels and background factors^19^, were prospectively followed up for 60 months. Each patient underwent HD therapy thrice a week for 3–4 h at the same time each day.

### Background factors

At the start of this study, we collected information on the study population, including age, sex, body mass index (BMI), primary disease (the presence or absence of diabetes mellitus), duration of HD, smoking status, past history of cardiovascular events and malignant diseases, urine volume (≥ 0 ml/day), and consumption of selected medications. BMI was calculated as follows: BMI = {*post-dialysis body weight (kg)/[height (m)]*^2^} × 100. Five systolic blood pressure (SBP) values were measured on the 1st dialysis day of the week: the first was SBP at the start of dialysis; the second and third were the highest and lowest SBP during dialysis, respectively; the fourth was the difference between the highest and lowest values; the fifth was SBP at the end of dialysis. The post-dialysis cardiothoracic ratio (CTR) values, which were the ratio of maximal horizontal cardiac diameter to maximal horizontal thoracic diameter (inner edge of ribs/edge of pleura) on posteroanterior chest x-ray measurements, were obtained on the 1st dialysis day of the week. The normalized dialysis dose (Kt/V) was calculated on the 1st dialysis day of the week using the following equation, i.e., the formula of Daugirdas^24^: Kt/V = − *Ln {[(post-dialysis value of blood urea nitrogen (BUN)/pre-dialysis value of BUN)* − *(0.008* × *dialysis time)]* + *[4 – (3.5* × *post-dialysis value of BUN/pre-dialysis value of BUN)]* × *(amount of drainage/post-dialysis body weight)}.*

### Blood examinations

Non-fasting blood samples were obtained while patients were lying in bed in a supine position after at least 15 min of rest on the 1st dialysis day of the week. The following pre-dialysis parameters were measured: hemoglobin (Hb), high-density lipoprotein cholesterol, low-density lipoprotein cholesterol, triglyceride (TG), albumin-corrected calcium, inorganic phosphorus, intact parathyroid hormone, plasma renin activity, plasma aldosterone concentration, aldosterone to renin ratio, HbA1c, and creatinine (Cre), uric acid, C-reactive protein (CRP), and albumin (Alb) levels. Pre-dialysis serum s(P)RR levels were measured using an enzyme-linked immunosorbent assay (ELISA) kit (Takara Bio Inc., Otsu City, Japan) consisting of a solid-phase sandwich ELISA with highly specific antibodies for each protein^25^.

The following post-dialysis values were measured using conventional methods at an external testing laboratory (Kishimoto, Inc., Tomakomai City, Japan): human atrial natriuretic peptide (hANP), a marker of body fluid volume^26–28^ and BNP.

### Physiological function tests

#### Echocardiography

Echocardiography was performed on a non-dialysis day, as previously described^29^, using the Vivid S6 System (GE Healthcare, Milwaukee, WI, USA), and cardiac functions were estimated as follows: (1) left ventricular ejection fraction, as a marker of contractile activity; (2) left ventricular mass index, as a marker of cardiac hypertrophy^30^; and (3) E/eʹ and deceleration time, as markers of left ventricular diastolic function^31^.

#### Carotid intima-media thickness

Ultrasonographic examinations of the common carotid artery, bulb, and internal carotid artery were bilaterally performed on a non-dialysis day, as described previously^32^, using the Nemio 30 Ultrasound System (Toshiba Medical Systems Co., Ltd, Tochigi, Japan).

#### Ankle-brachial index and brachial-ankle pulse wave velocity

The ankle-brachial index (ABI) (average and lower values) and brachial-ankle pulse wave velocity (baPWV) values (average and higher values) were measured on a non-dialysis day using a volume-plethysmographic apparatus PWV/ABI (Omron Healthcare Co., Ltd., Kyoto, Japan) following previously described methods^33^. The BaPWV cannot be properly estimated when the ABI is lower than 0.9 because arterial occlusion retards the baPWV^34,35^. Therefore, patients with ABI < 0.9 were excluded from the baPWV analysis.

### Computed tomography

Body fat distribution was determined on a non-dialysis day using computed tomography (CT) imaging with a 64-row multislice CT scanner (Aquilion 64; Toshiba, Tokyo, Japan). The subcutaneous and visceral fat areas were measured at the level of the umbilicus using the Ziostation 2 software (Ziosoft, Tokyo, Japan).

### New-onset cardiovascular events or malignant diseases

The occurrence of cardiovascular events or malignant diseases was investigated by either verifying with the electronic medical record for patients who had been undergoing dialysis at our affiliated clinic for five years (226 patients out of 258) or through telephone or letter (32 patients). Cardiovascular events were defined as (1) cardiovascular death, (2) nonfatal myocardial infarction, (3) unstable angina, (4) heart failure, (5) cerebral infarction or cerebral hemorrhage, and (6) severe lower limb ischemia (severe arteriosclerosis obliterans). Malignant diseases include stomach, colorectal, small intestinal, lung, renal, bladder, ureteral, penile, laryngeal, thyroid gland, and breast cancers, uterine neoplasms, and malignant melanomas.

### Study protocols

The pre-dialysis serum s(P)RR levels on the 1st dialysis day of the week were measured at the start of the study. The patients were divided into two groups (higher and lower groups) according to the median serum s(P)RR levels, and the two groups were compared in terms of background factors and blood, physiological function, and CT data. Patients were followed up for 60 months or until death from any cause, and the relationship between serum s(P)RR levels and new-onset cardiovascular events or malignant diseases was investigated using annual physiological tests, such as abdominal echo and CT imaging. In addition, the association between serum s(P)RR values and all-cause, cardiovascular, and non-cardiovascular mortalities was investigated between the two groups.

### Statistical analyses

Normally distributed continuous variables are expressed as means ± standard deviations and non-normally distributed ones as medians with interquartile ranges (25th and 75th percentiles). Intergroup comparisons of parameters were performed using the Wilcoxon signed-rank or Mann–Whitney U test. Categorical variables are presented as the number of patients and compared using the chi-square test. Kaplan–Meier plots and log-rank tests were also used to compare the all-cause, cardiovascular, and non-cardiovascular mortalities between the two groups (higher versus lower serum s(P)RR values). The C-index and area under the curve values were used to evaluate the effects of specific variables on survival^36^. Background factors contributing to cardiovascular mortality were analyzed using univariate Cox proportional hazard regression. In addition, we constructed multivariate Cox proportional hazard regression models to estimate the hazard ratios (HR) and 95% confidence intervals (95% CI) for cardiovascular mortality using factors showing significant correlations as covariates with mortality. The level of significance was set at *P* < 0.05. All analyses were performed using the Bell Curve for Excel (Social Survey Research Information Co. Ltd., Tokyo, Japan).

## Results

### Characteristics of the study patients at baseline

All 255 patients, excluding three who received renal transplants during the study period, could be consecutively followed up for 60 months or until death [60 (38–60)]. The duration of HD therapy ranged from 1 month to a maximum of 360 months and the median was 48 (24–83) months. The median serum s(P)RR value of these patients at baseline was 29.8 ng/ml. Table 1 details the baseline characteristics of the study patients in the two groups (higher group, serum s(P)RR level ≥ 29.8 ng/ml; lower group, < 29.8 ng/ml) and includes background factors and blood, physiological function, and CT data.

**Table 1:** Comparison of characteristics of the study participants at baseline between the higher and lower serum s(P)RR concentration groups.

	Higher group	Lower group	*P* value
	(n = 129)	(n = 129)	
**Background factors**			
Age (years)	72 (62–77)	68 (59–74)	0.07
Sex (male/female)	71/58	75/54	0.615
Body mass index (kg/m^2^)	21.8 (19.9–24)	21.6 (19.4–24.2)	0.549
Primary disease (DM/non-DM)	55/74	68/61	0.105
Duration of hemodialysis therapy (months)	48 (24–84)	48 (24–72)	0.788
Smoking history (yes/no)	55/74	60/69	0.531
Past history of cardiovascular events (yes/no)	58/71	63/66	0.266
Past history of malignant diseases (yes/no)	15/114	24/105	0.059
Urine volume (0 ml/day or more than 0 ml/day)	39/90	28/101	0.94
**SBP during dialysis (mmHg)**			
At the beginning	145 ± 20	154 ± 18	< 0.001
Highest values	156 ± 21	164 ± 20	< 0.001
Lowest values	121 ± 20	128 ± 21	0.008
Difference (the highest–the lowest)	32 (24–42)	30 (22–46)	0.194
At the end	136 ± 20	143 ± 22	0.002
Medications (yes/no)			
RAS-I	89/40	108/21	0.005
β-blocker	44/85	54/75	0.217
CCB	90/39	100/29	0.188
Statin	17/112	18/111	0.428
Phosphate binder	106/23	106/26	0.5
CTR (%)	53.0 (49.4–55.4)	51.7 ± 5.2	0.04
Kt/V	1.36 ± 0.26	1.34 ± 0.22	0.313
**Blood tests**			
Hemoglobin (g/dl)	10.9 ± 1.0	10.7 (10.2–11.2)	0.036
HDL cholesterol (mg/dl)	46.0 (38.0–55.0)	45.0 (36.0–55.0)	0.632
LDL cholesterol (mg/dl)	91.3 ± 30.5	83.0 (64.0–103.0)	0.203
Triglycerides (mg/dl)	96.0 (68.0–136.0)	81.0 (55.0–113.0)	0.004
Albumin-corrected calcium (mg/dl)	8.8 (8.5–9.2)	8.8 (8.6–9.1)	0.97
Inorganic phosphorus (mg/dl)	5.4 (4.4–6.0)	5.1 ± 1.1	0.064
Intact parathyroid hormone (pg/ml)	139.0 (84.0–185.0)	123.0 (76.0–176.0)	0.305
Creatinine (mg/dl)	9.5 ± 2.5	9.7 ± 2.6	0.478
Uric acid (mg/dl)	7.3 ± 1.4	7.0 ± 1.3	0.053
CRP (mg/dl)	0.13 (0.05–0.49)	0.07 (0.05–0.19)	< 0.001
Albumin (g/dl)	3.7 ± 0.3	3.7 (3.5–3.9)	0.422
hANP (pg/ml)	48.2 (28.2–78.2)	46.7 (31.5–74.9)	0.866
BNP (pg/ml)	136.6 (76.4–287.4)	148.9 (74.9–244.5)	0.974
PRA (ng/ml)	1.7 (0.8–4.0)	2.3 (0.8–4.2)	0.438
PAC (pg/ml)	8.8 (6.6–12.6)	7.5 (6.5–10.2)	0.673
ARR (PAC/PRA)	5.7 (2.3–13.0)	3.7 (2.0–11.7)	0.828
HbA1c (%)	5.0 (4.7–5.5)	5.2 (4.9–5.7)	0.976
**Physical function tests**			
Echocardiography			
LVEF (%)	67.6 (62.8–72.1)	67.2 (63.5–71.5)	0.361
LVMI (g/m^2^)	171.1 (144.9–202.7)	180.5 ± 50.0	0.202
E/e'	18.0 (14.0–22.1)	18.8 (14.9–22.2)	0.404
Dec-T	229.0 (199.8–264.1)	231.7 ± 54.1	0.817
Carotid ultrasound examination			
Max carotid IMT (mm)	0.9 (0.7–1.0)	0.9 (0.7–1.1)	0.394
ABI			
≥ 0.9/ < 0.9	96/25	107/19	0.252
Average values	1.15 (1.05–1.23)	1.17 (1.07–1.24)	0.378
Lower values	1.13 (0.95–1.20)	1.13 (1.00–1.21)	0.776
baPWV (cm/s)			
Average values	1794.8 (1568.4–2065.9)	1853.5 (1606.8–2176.8)	0.719
Higher values	1907.0 (1639.5–2165.8)	1921.0 (1641.5–2234.0)	0.774
Abdominal CT			
Subcutaneous fat area (cm^2^)	110.9 (72.2–171.2)	105.5 (59.8–155.6)	0.105
Visceral fat area (cm^2^)	79.1 (47.7–119.3)	69.4 (33.1–112.1)	0.097

Intergroup comparisons of parameters without correspondence were performed using the Mann–Whitney U test and those with correspondence were performed using the Wilcoxon signed-rank test. In addition, categorical variables have been presented as the number of patients and compared using the chi-square test. s(P)RR, soluble (pro)renin receptor; DM, diabetes mellitus; SBP, systolic blood pressure; RAS-I, renin-angiotensin system inhibitor; CCB, calcium channel blocker; CTR, cardiothoracic ratio; Kt/V, normalized dialysis dose; HDL cholesterol, high-density lipoprotein cholesterol; LDL cholesterol, low-density lipoprotein cholesterol; CRP, C-reactive protein; hANP, human atrial natriuretic peptide; BNP, brain natriuretic peptide; PRA, plasma renin activity; PAC, plasma aldosterone concentration; ARR, aldosterone to renin ratio; LVEF, left ventricular ejection fraction; LVMI, left ventricular mass index; E/e', E over e-prime; Dec-T, deceleration time; IMT, intima-media thickness; ABI, ankle-brachial index; baPWV, brachial-ankle pulse wave velocity; CT, computed tomography.

The number of patients with ABI measurements was 247 (96.9%). ABI was < 0.9 in 44 patients and as a result 203 patients underwent the baPWV analysis. When comparing between higher and lower groups of serum s(P)RR concentration, the CTR, Hb, TG, and CRP levels were significantly greater, and the highest and lowest SBP values and those at the beginning and end of the dialysis were significantly smaller in the higher group than in the lower group. The percentage of patients taking RAS-inhibitors (RAS-Is), such as angiotensin receptor blockers, angiotensin-converting enzyme inhibitors, and renin inhibitors, was significantly greater in the lower group than in the higher group (83.7% vs. 69.0%). Primary disease (presence or absence of diabetes mellitus) was not significantly different between the two groups. In addition, the BMI and Kt/V did not significantly differ between the two groups, suggesting that HD parameters may not strongly influence the serum s(P)RR levels. There were 121 (47.4%) patients with a past history of cardiovascular events; 13 (4.7%) had nonfatal myocardial infarction, 41 (16.1%) unstable angina, 29 (11.4%) heart failure, 56 (22.0%) cerebral infarction or cerebral hemorrhage, and 14 (5.5%) severe lower limb ischemia (severe arteriosclerosis obliterans). There were 27 (10.6%) patients with a past history of multiple cardiovascular events. Thirty-nine patients (15.1%) had a past history of malignant diseases; 10 (3.9%) had stomach cancer, eight (3.1%) colorectal cancer, one (0.4%) small intestinal cancer, four (1.6%) lung cancer, six (2.3%) renal cancer, four (1.6%) bladder cancer, one (0.4%) ureteral cancer, one (0.4%) penile cancer, one (0.4%) laryngeal cancer, one (0.4%) thyroid gland cancer, three (1.2%) breast cancer, one (0.4%) uterine neoplasm, and one (0.4%) malignant melanoma. There were two patients (0.8%) with a past history of multiple malignant diseases. The ratios of the past histories of cardiovascular events and malignant diseases were not significantly different between the groups.

### Estimating the risk of new-onset cardiovascular events

**Figure 1:**
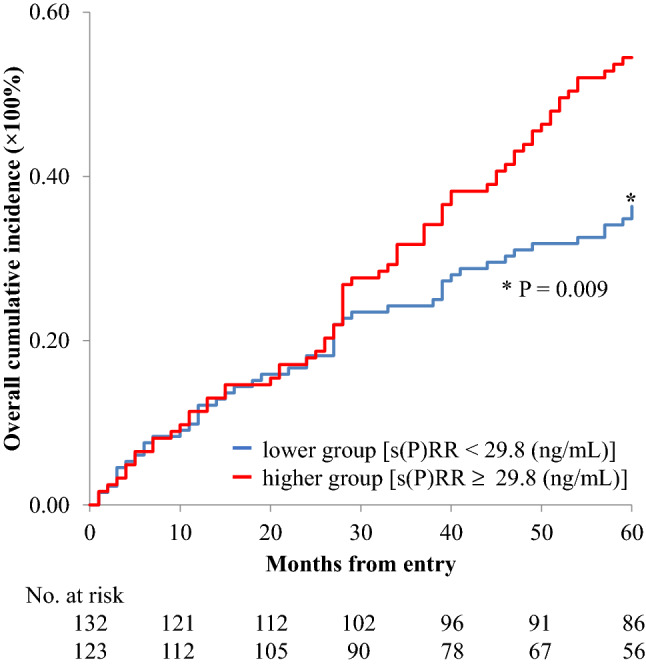
Cumulative probability of cardiovascular events. Kaplan–Meier plots comparing patients with higher and lower serum concentrations of s(P)RR. s(P)RR, soluble (pro)renin receptor.

During the 60-month follow-up period, 115 patients recorded new-onset cardiovascular events; 27 died of cardiovascular causes (23.5%), 5 had nonfatal myocardial infarction (4.3%), 26 unstable angina (22.6%), 12 heart failure (10.4%), 24 cerebral infarction or cerebral hemorrhage (20.9%), and 21 severe lower limb ischemia (severe arteriosclerosis obliterans) (18.3%). The serum s(P)RR levels were significantly higher in patients with new-onset cardiovascular events (31.2 ± 6.1 ng/ml, n = 115) than in those without them (29.8 ± 6.1 ng/ml, n = 140); *P* = 0.039. The serum s(P)RR levels in patients who developed severe lower limb ischemia were particularly high (Table 2). The incidence of new-onset cardiovascular events at the end of the follow-up period was significantly higher in patients with higher serum s(P)RR levels (52.8%) than in those with lower serum s(P)RR levels (38.3%); *P* = 0.010. The cumulative incidence of new-onset cardiovascular events was significantly greater in patients with higher serum s(P)RR concentrations than in those with lower s(P)RR concentrations (Fig. 1).

**Table 2:** Average of serum s(P)RR levels in patients with each new-onset cardiovascular event.

	n	s(P)RR levels (ng/ml)
Cardiovascular death	27	31.5 (29.6–34.9)
Nonfatal myocardial infarction	5	29.6 (26.5–31.1)
Unstable angina	26	29.1 (25.6–32.4)
Heart failure	12	31.7 (29.2–33.6)
Cerebral infarction or cerebral hemorrhage	24	30.6 ± 7.2
Severe lower limb ischemia (severe arteriosclerosis obliterans)	21	34.1 ± 5.8

s(P)RR, soluble (pro)renin receptor.

### Estimating the risk of new-onset malignant diseases

During the 60-month follow-up period, 38 patients recorded new-onset malignant diseases. At the end of the follow-up period, the serum s(P)RR levels did not significantly differ between patients with new-onset malignant diseases [30.5 (26.5–34.4) ng/ml, n = 38] and those without (30.4 ± 6.2 ng/ml, n = 217); *P* = 0.420. There was no difference in serum s(P)RR levels depending on the malignant-disease type (Table 3). There was no difference in the incidence of new-onset malignant diseases between patients with higher serum s(P)RR levels (16.5%) and those with lower levels (15.6%); *P* = 0.422.

**Table 3:** Average of serum s(P)RR levels in patients with each new-onset malignant disease.

	n	s(P)RR levels (ng/ml)
**Gastrointestinal cancer**		
Stomach cancer	7	32.2 (29.0–37.6)
Colorectal cancer	5	29.6 (24.8–34.9)
Esophageal cancer	1	31.7
Appendiceal cancer	2	35.8
**Liver, gallbladder, and pancreatic cancer**		
Hepatoma	2	30.1
Respiratory cancer		
Lung cancer	6	27.3 (21.2–30.3)
Malignant pleural mesothelioma	1	34.4
Urinary organ cancer		
Renal cancer	5	33.0 (26.1–33.8)
Bladder cancer	3	27.4 (26.4–29.7)
Prostate cancer	3	28.1 (24.7–32.3)
**Others**		
Hypopharyngeal cancer	1	27.3
Breast cancer	2	28.1
Malignant melanoma	1	31.7

s(P)RR, soluble (pro)renin receptor.

### Association between serum s(P)RR levels and prognosis

During the 60-month follow-up period, 106 deaths (41.6%) were recorded, including 63 cardiovascular deaths (24.7%)—2 due to acute myocardial infarction, 27 due to congestive heart failure, 15 due to lethal arrhythmia, and 6 due to cerebral hemorrhage, and 13 sudden unexpected deaths. Forty-three non-cardiovascular deaths (16.9%) were recorded—15 due to infectious diseases, 21 due to cachexia, 5 due to cancer, and 2 due to liver failure. The 1-year, 2-year, 3-year, 4-year, and 5-year survival rates were 91.8%, 85.5%, 76.1%, 69.0%, and 58.0%, respectively. At the end of the follow-up period, the serum s(P)RR levels were significantly higher in patients who died by any cause (31.8 ± 5.8 ng/ml, n = 106) and in those who died of cardiovascular causes (32.5 ± 6.5 ng/ml, n = 63) than in those who survived (29.5 ± 6.3 ng/ml, n = 149 and 29.7 ± 5.8 ng/ml, n = 192, respectively; *P* = 0.005, < 0.001, respectively). The higher serum s(P)RR group had higher rates of total (49.6%) and cardiovascular (33.9%) deaths than the lower serum s(P)RR group (total: 33.6% and cardiovascular: 15.6%, respectively) (*P* = 0.005 and *P* < 0.001, respectively).

**Figure 2:**
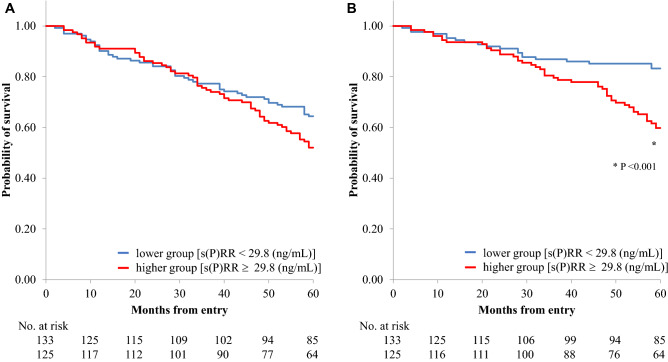
Probability of survival (**A**, All-cause death; **B**, Cardiovascular death). Kaplan–Meier survival plots comparing patients with higher and lower serum concentrations of s(P)RR. s(P)RR, soluble (pro)renin receptor.

**Figure 3:**
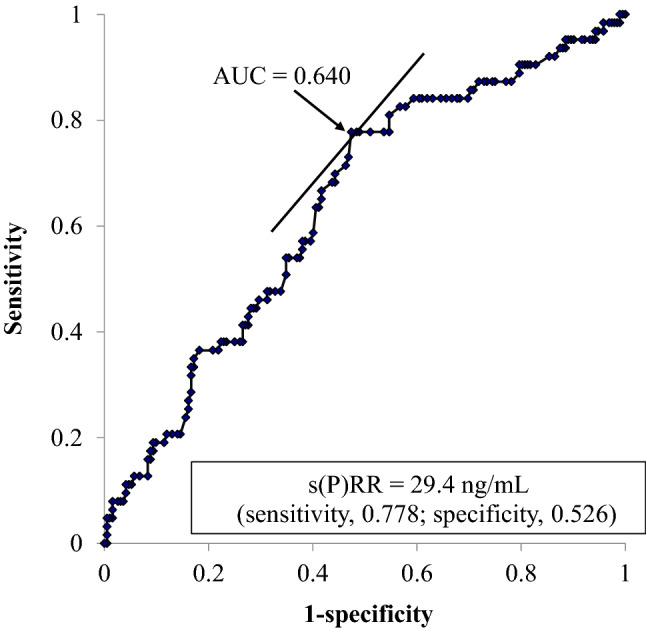
Receiver operating characteristic curve with area under the curve values. Discrimination value of s(P)RR for the survival rate using receiver operating characteristic analysis. AUC, area under the curve; s(P)RR, soluble (pro)renin receptor.

Kaplan–Meier analyses showed that the cumulative survival rate of total death in the higher serum s(P)RR group was not significantly lower than that in the lower group (log-rank test, χ2 = 3.0, *P* = 0.083), but the cumulative survival rate of cardiovascular death in the higher group was significantly lower than that in the lower group (log-rank test, χ2 = 11.1, *P* < 0.001) (Fig. 2). A receiver operating characteristic curve was constructed to determine the optimal cutoff value of the serum s(P)RR level for cardiovascular death (Fig. 3). The optimal cutoff value of the serum s(P)RR level for cardiovascular death was 29.4 ng/ml (sensitivity, 0.778; specificity, 0.526) (Fig. 3). Univariate Cox regression analyses showed that age, primary disease (diabetes mellitus) status; the difference (the highest – the lowest) in SBP values; medications (statin); CTR, s(P)RR, TG, hANP, BNP, and HbA1c values; max carotid intima-media thickness; and baPWV (average and higher values) were significantly and positively correlated while SBP (highest values), SBP (lowest values), SBP values at the end, medications (RAS-I), medications (calcium channel blockers), Cre, Alb, and ABI (average and lower values) were significantly and negatively correlated with cardiovascular death (Table 4). The results of the multivariate Cox regression analyses for cardiovascular mortality are shown in Table 5. All factors that were significantly correlated with total death in the univariate analyses (Table 4) were used as covariates in **model 1**. Factors that correlated with cardiovascular death, excluding ABI and baPWV, were used as covariates in **model 2**. Although the s(P)RR level was not significantly correlated with cardiovascular death in **model 1**, it showed a significant positive relationship with cardiovascular death in **model 2** (HR 1.041, 95% CI 1.001–1.083, *P* = 0.046).

**Table 4:** Univariate Cox regression analyses of variables for cardiovascular deaths.

Variable	HR	95% CI	*P* value
**Background factors**			
Age	1.055	1.027–1.083	< 0.001
Sex (male)	N.S	N.S	N.S
Body mass index	N.S	N.S	N.S
Primary disease (DM)	1.778	1.076–2.935	0.025
Duration of hemodialysis therapy	N.S	N.S	N.S
Smoking history (yes)	N.S	N.S	N.S
Urine volume (more than 0 ml/day)	N.S	N.S	N.S
SBP during dialysis			
At the beginning	0.988	0.974–1.001	0.07
Highest values	0.986	0.973–0.999	0.032
Lowest values	0.942	0.960–0.985	< 0.001
Difference (the highest–the lowest)	1.012	1.001–1.023	0.028
At the end	0.976	0.964–0.988	< 0.001
Medications (yes)			
RAS-I	0.478	0.283–0.808	0.006
β-blocker	N.S	N.S	N.S
CCB	0.504	0.302–0.842	0.009
Statin	2.35	1.332–4.146	0.003
Phosphate binder	N.S	N.S	N.S
CTR	1.103	1.052–1.155	< 0.001
Kt/V	N.S	N.S	N.S
**Blood tests**			
s(P)RR	1.057	1.020–1.095	0.003
Hemoglobin	N.S	N.S	N.S
HDL cholesterol	0.982	0.965–1.000	0.054
LDL cholesterol	N.S	N.S	N.S
Triglycerides	1.004	1.001–1.007	0.024
Calcium	N.S	N.S	N.S
Inorganic phosphorus	N.S	N.S	N.S
Intact parathyroid hormone	1.002	0.999–1.004	0.178
Creatinine	0.884	0.805–0.972	0.01
Uric acid	N.S	N.S	N.S
CRP	1.184	0.916–1.530	0.197
Albumin	0.324	0.142–0.741	0.008
hANP	1.003	1.002–1.004	< 0.001
BNP	1.001	1.001–1.002	< 0.001
PRA	N.S	N.S	N.S
PAC	N.S	N.S	N.S
ARR (PAC/PRA)	N.S	N.S	N.S
HbA1c	1.339	1.044–1.716	0.022
**Physical function tests**			
Echocardiography			
LVEF	0.976	0.951–1.001	0.064
LVMI	N.S	N.S	N.S
E/e'	N.S	N.S	N.S
Dec-T	N.S	N.S	N.S
Carotid ultrasound examination			
Max carotid IMT	1.952	1.219–3.127	0.005
ABI			
Average values	0.249	0.072–0.861	0.028
Lower values	0.246	0.084–0.719	0.01
baPWV			
Average values	1.001	1.000–1.001	0.045
Higher values	1.001	1.000–1.001	0.013
Abdominal CT			
Subcutaneous fat area	N.S	N.S	N.S
Visceral fat area	N.S	N.S	N.S

HR, hazard ratio; CI, confidence intervals; N.S., not selected as a potential covariate; s(P)RR, soluble (pro)renin receptor; DM, diabetes mellitus; SBP, systolic blood pressure; RAS-I, renin-angiotensin system inhibitor; CCB, calcium channel blocker; CTR, cardiothoracic ratio; Kt/V, normalized dialysis dose; HDL cholesterol, high-density lipoprotein cholesterol; LDL cholesterol, low-density lipoprotein cholesterol; CRP, C-reactive protein; hANP, human atrial natriuretic peptide; BNP, brain natriuretic peptide; PRA, plasma renin activity; PAC, plasma aldosterone concentration; ARR, aldosterone to renin ratio; LVEF, left ventricular ejection fraction; LVMI, left ventricular mass index; E/e', E over e-prime; Dec-T, deceleration time; IMT, intima-media thickness; ABI, ankle-brachial index; baPWV, brachial-ankle pulse wave velocity; CT, computed tomography.

**Table 5:** Multivariate Cox regression analyses of variables for cardiovascular deaths.

Variable	Model 1			Model 2		
	Including ABI, baPWV			Excluding ABI, baPWV		
	HR	95% CI	*P* value	HR	95% CI	*P* value
s(P)RR	N.S	N.S	N.S	1.041	1.001–1.083	0.046
Age	1.039	1.004–1.074	0.027	1.048	1.017–1.079	0.002
Primary disease (DM)	N.S	N.S	N.S	1.900	1.101–3.278	0.021
Highest SBP values	N.S	N.S	N.S	N.S	N.S	N.S
Lowest SBP values	0.970	0.955–0.985	< 0.001	0.981	0.969–0.993	0.002
The difference in SBP values (the highest–the lowest)	N.S	N.S	N.S	N.S	N.S	N.S
SBP values at the end	N.S	N.S	N.S	N.S	N.S	N.S
Medications (RAS-I)	N.S	N.S	N.S	N.S	N.S	N.S
Medications (CCB)	N.S	N.S	N.S	N.S	N.S	N.S
Medications (statin)	2.555	1.190–5.485	0.016	2.975	1.544–5.730	0.001
CTR	1.083	1.021–1.148	0.008	1.057	1.005–1.113	0.033
Triglycerides	N.S	N.S	N.S	1.003	0.999–1.008	0.159
Creatinine	N.S	N.S	N.S	1.106	0.974–1.256	0.120
Albumin	N.S	N.S	N.S	N.S	N.S	N.S
hANP	N.S	N.S	N.S	N.S	N.S	N.S
BNP	1.001	1.001–1.002	< 0.001	1.001	1.001–1.001	< 0.001
HbA1c	N.S	N.S	N.S	N.S	N.S	N.S
ABI (average values)	N.S	N.S	N.S	–	–	–
ABI (lower values)	N.S	N.S	N.S	–	–	–
BaPWV (average values)	N.S	N.S	N.S	–	–	–
BaPWV (higher values)	1.001	1.000–1.001	0.009	–	–	–
Max carotid IMT	0.354	0.121–1.038	0.059	N.S	N.S	N.S

ABI, ankle-brachial index; baPWV, brachial-ankle pulse wave velocity; HR, hazard ratio; CI, confidence interval; s(P)RR, soluble (pro)renin receptor; N.S., not selected as a potential covariate; DM, diabetes mellitus; SBP, systolic blood pressure; RAS-I, renin-angiotensin system inhibitor; CCB, calcium channel blocker; CTR, cardiothoracic ratio; CRP, C-reactive protein; hANP, human atrial natriuretic peptide; BNP, brain natriuretic peptide; IMT, intima-media thickness.

## Discussion

The present study investigated the relationship between high serum s(P)RR levels and the occurrence of cardiovascular events or malignant diseases and the prognosis in patients undergoing HD; the study produced three major findings. First, the CTR, Hb, TG, and CRP levels were significantly higher, and several SBP values were significantly lower in the higher serum s(P)RR group than in the lower serum s(P)RR group. Second, the occurrence ratio of cardiovascular events was significantly higher in the higher serum s(P)RR group than in the lower serum s(P)RR group. Finally, the serum s(P)RR level was independently associated with cardiovascular mortality, suggesting that increased expression of (P)RR may be associated with the progression of cardiovascular events in patients undergoing HD.

In the present study, atherogenic factors, such as the CTR, TG, and CRP levels, were found to be greater in the higher serum s(P)RR group than in the lower serum s(P)RR group (Table 1) in accordance with the findings of our previous report^19^.

We previously reported that the serum s(P)RR level was associated with atherosclerosis, independent of other risk factors, in patients undergoing HD^19^, and a high serum s(P)RR level was associated with an increase in BNP, independent of other risk factors^21^. From a clinical perspective, these considerations suggest that patients with elevated s(P)RR concentrations should be screened for CVD risk factors. Conversely, there was no difference between the higher and lower s(P)RR groups with respect to their past histories of CVD or malignant diseases (Table 1). These results suggest that the serum s(P)RR concentration at the time of measurement may not be affected by the past history of cardiovascular events or malignant diseases.

In this study, the serum s(P)RR levels were significantly higher in patients who experienced cardiovascular events during the follow-up period than in patients who did not; vice versa the incidence of cardiovascular events was significantly higher in patients with higher serum s(P)RR levels than in those with lower serum s(P)RR levels (Fig. 1). We previously reported that high serum s(P)RR levels in patients undergoing HD were associated with severe atherosclerosis of the lower limbs, independent of other risk factors^19^. Therefore, we considered it is possible that serum s(P)RR could be used as a marker for atherosclerotic conditions. We also reported that a high serum s(P)RR level was associated with an increase in BNP during the first 1-year follow-up period, independent of other risk factors^21^, suggesting that increased expression of (P)RR may be associated with the progression of heart failure in patients undergoing HD. Accordingly, higher s(P)RR levels may be associated with increased occurrence of cardiovascular events in patients undergoing HD, and serum s(P)RR could be potentially used as a predictive marker for cardiovascular events in these patients.

(P)RR acts as an adaptor protein that co-locates with the Wnt receptor complex and contributes to the activation of Wnt/β-catenin signaling, independent of RAS^8^. The Wnt/β-catenin signaling pathway plays a pivotal role in numerous biological processes, such as in embryonic development, tissue homeostasis, and carcinogenesis^9^. Overexpression of (P)RR has been observed in various malignant diseases, including pancreatic^13,37^, brain^14,38^, colorectal^39,40^, breast^12^, adrenal^15^, and endometrial^41^ cancers. Overexpression of (P)RR may contribute to cancer initiation and progression via the Wnt/β-catenin, RAS, mitogen-activated protein kinase/extracellular signal-regulated kinase (ERK), and phosphatidylinositol 3-kinase-protein kinase/protein kinase B/mammalian target of rapamycin pathways, as well as to V-ATPase function in various cancers^42^.

Patients undergoing HD are at a high risk for malignant diseases in the kidneys, bladder, thyroid, and other endocrine organs^22,23^. Although the reason for the increased risk of malignant diseases in patients undergoing HD remains unclear, there are several possible explanations, including chronic inflammation, inhibition of the immune system^43^, poor nutritional status, reduced antioxidant capacity, accumulation of carcinogens^44^, and dialysis-related factors^45^. In the present study, however, there was no difference in the incidence of malignant diseases between patients with higher and lower serum s(P)RR levels (Table 3). The possible reasons for this lack of association between s(P)RR concentration and cancer are insufficient sample size and limited follow-up. Besides, we cannot exclude the possibility of other confounding factors that affect the occurrence of malignant diseases in patients undergoing HD masking the effects of (P)RR. This issue should be addressed in further investigations.

Chronic kidney disease (CKD) is associated with the risk of developing CVD^46^, and the risk of total death, cardiovascular death, and hospitalization due to CVDs is high in patients with CKD^47^. In keeping with these data, the present study showed high all-cause mortality (41.6%) and high cardiovascular mortality (24.7%) in patients undergoing HD during the 60-month follow-up period. In patients undergoing HD, age, primary disease (diabetes mellitus) status, intradialytic BP change^48^, intradialytic hypotension^49^, serum Alb level^50^, and medication (RAS-I)^51^ are, in general, associated with prognosis. In line with these reports, our study showed that age, primary disease (diabetes mellitus) status, and intradialytic BP change were significantly and positively correlated and the lowest intradialytic BP, medications (RAS-Is), and Alb level were significantly and negatively correlated with cardiovascular deaths (Table 4). Although the cumulative survival rate of cardiovascular death in the higher s(P)RR group was significantly lower than that in the lower s(P)RR group (Fig. 2), the difference was not apparent until 20 months after the initiation of the study. The reason for this remains unclear; however, as the two groups had similar cardiac functions, carotid intima-media thicknesses, ABI and baPWV values, and histories of CVD at the start of this study, sufficient time may have been required for the differences to manifest (Table 1).

This study showed for the first time that the s(P)RR level is associated with cardiovascular mortality in patients undergoing HD. Atherosclerosis and vascular calcification have been shown to be risk factors for CVD in patients with CKD^52,53^. (P)RR-mediated ERK signal transduction, independent of the generation of angiotensin II or the activation of its receptor, contributed to the development of vascular complications^54,55^. The serum s(P)RR concentration was associated with arteriosclerosis^19^ and worsening of heart failure^21^. We also showed that the long-term administration of a (P)RR blocker attenuated the development of cardiac fibrosis and hypertrophy^56^. Therefore, although the mechanism by which the blood s(P)RR concentration is associated with cardiovascular mortality remains unclear, we suppose that a high s(P)RR concentration could be associated with cardiovascular mortality via increased tissue (P)RR expression and atherosclerosis and/or heart failure and subsequent cardiovascular events. Further studies are required to test this assumption.

Several limitations of the present study warrant mention. First, our sample size was relatively small. Second, the present data from patients undergoing HD may have been modulated by HD therapy because s(P)RR may have been dialyzed to some extent^14^. Third, the mechanisms by which the serum s(P)RR level is associated with background factors remain unclear. Further studies are required to clarify the role of serum s(P)RR in patients undergoing HD. Fourth, we measured serum s(P)RR levels only at baseline. Serum s(P)RR levels may change with time depending on the conditions of (P)RR expressing organs such as the heart, brain, and pancreas^1^. Therefore, repeated measurements of serum s(P)RR levels with time by future studies might reveal further findings.

In conclusion, high serum s(P)RR level in patients undergoing HD was associated with various background factors and the occurrence of cardiovascular events. Furthermore, a high serum s(P)RR level was independently correlated with cardiovascular mortality. Therefore, s(P)RR could potentially be used as a marker for the occurrence of cardiovascular mortality and thus, could be useful in selecting patients requiring intensive care. Further studies are needed to determine whether reducing the serum s(P)RR level would improve patient prognosis in this population.
